# HIV coinfection influences the inflammatory response but not the outcome of cerebral malaria in Malawian children

**DOI:** 10.1016/j.jinf.2016.05.012

**Published:** 2016-09

**Authors:** Emmie W. Mbale, Christopher A. Moxon, Mavuto Mukaka, Maganizo Chagomerana, Simon Glover, Ngawina Chisala, Sofia Omar, Malcolm Molyneux, Karl Seydel, Alister G. Craig, Terrie Taylor, Robert S. Heyderman, Macpherson Mallewa

**Affiliations:** a Malawi-Liverpool-Wellcome Trust Clinical Research Programme, University of Malawi College of Medicine, Malawi; b Department of Paediatrics, University of Malawi College of Medicine, Malawi; c Liverpool School of Tropical Medicine, UK; d Institute of Infection and Global Health, University of Liverpool, UK; e Blantyre Malaria Project, University of Malawi College of Medicine, Malawi; f College of Osteopathic Medicine, Michigan State University, East Lansing, MI, United States; g School of Medicine, University of St. Andrews, UK; h University College London, UK

**Keywords:** Cerebral malaria, HIV, Paediatric, TNF

## Abstract

**Objectives:**

Study of the effect of HIV on disease progression in heterogeneous severe malaria syndromes with imprecise diagnostic criteria has led to varying results. Characteristic retinopathy refines cerebral malaria (CM) diagnosis, enabling more precise exploration of the hypothesis that HIV decreases the cytokine response in CM, leading to higher parasite density and a poor outcome.

**Methods:**

We retrospectively reviewed data on clinical progression and laboratory parameters in 877 retinopathy-positive CM cases admitted 1996–2011 (14.4% HIV-infected) to a large hospital in Malawi. Admission plasma levels of TNF, interleukin-10, and soluble intercellular adhesion molecule (sICAM-1) were measured by ELISA in 135 retinopathy-positive CM cases.

**Results:**

HIV-infected CM cases had lower median plasma levels of TNF (p = 0.008), interleukin-10 (p = 0.045) and sICAM-1 (p = 0.04) than HIV-uninfected cases. Although HIV-infected children were older and more likely to have co-morbidities, HIV-status did not significantly affect parasite density (p = 0.90) or outcome (24.8% infected, vs. 18.5% uninfected; p = 0.13).

**Conclusion:**

In this well-characterised CM cohort, HIV-coinfection was associated with marked blunting of the inflammatory response but did not affect parasite density or outcome. These data highlight the complex influence of HIV on severe malaria and bring into question systemic inflammation as a primary driver of pathogenesis in human CM.

## Highlights

• Characteristic malaria retinopathy refined the diagnosis of cerebral malaria.
• HIV-infected cases had markedly lower TNF, IL10 and ICAM-1 than uninfected cases.
• HIV-infected children were older and more likely to have comorbidities.
• Peripheral parasite count, HRP2 and mortality were unaffected by HIV status.

## Introduction

In sub-Saharan Africa over 3 million children are infected with the Human Immunodeficiency Virus (HIV).^1^ There are in excess of 100 million cases of *Plasmodium falciparum* infection per year, leading to approximately half a million deaths, mainly in African children. While the overlap between the two diseases is considerable, with many malaria infections occurring in HIV-positive children,^2^ determining the effect of HIV on the severity and outcome of malaria has been problematic, leading to variable and apparently contradictory results.^3^, ^4^, ^5^, ^6^ Some studies have found increased parasite density, an association with more severe malaria and worse outcome, and others have not (See Table 1 for a summary of published literature). We propose that at least in part, the use of insufficiently stringent diagnostic criteria for cerebral malaria (CM), could have led to misclassification of cases and therefore variability in the associations identified.

CM is a prominent severe malaria syndrome defined by the WHO as unrousable coma (Blantyre Coma Score^7^ ≤2) in the presence of *P. falciparum* parasitaemia, with no other cause of coma found.^8^, ^9^ In the absence of additional criteria this clinical definition leads to over diagnosis of CM, leaving uncertainty as to whether coma is truly caused by parasitaemia or whether a person has an uncomplicated malaria infection and coma due to another aetiology. This is particularly problematic in high transmission settings where a high proportion of apparently well children in the community are parasitaemic. This was highlighted by a study at our centre in Malawi where a quarter of children diagnosed as having WHO-defined CM were found to have a non-malaria cause of coma and death at autopsy in the context of a peripheral parasitaemia.^9^ This mis-classification may be exacerbated by HIV co-infection which may increase the risk of other non-malarial co-morbidities causing coma and thus confound the ability to detect true associations between HIV, CM and outcome (e.g. peripheral parasite density, the inflammatory response or mortality).

Characteristic retinal changes that are indicative of sequestration of *P. falciparum*-infected red blood cells (iRBC) in the neurovasculature^10^ distinguish with high specificity and sensitivity those children with histological evidence of CM, from those with a non-malarial coma.^9^ In order to re-examine the impact of HIV on CM, we have therefore used this refined diagnosis to classify a large cohort of Malawian children with CM, with and without HIV co-infection. Following the observation that peripheral blood mononuclear cells from HIV-infected individuals have impaired tumour necrosis factor-alpha (TNF) and interleukin 10 (IL-10) production *in vitro* in response to iRBC challenge,^11^ we addressed the specific hypothesis that HIV-infection results in lower levels of systemic TNF and IL-10 in CM *in vivo* and that this is associated with a higher peripheral parasite density and a higher mortality.

## Methods

### Location

This study was conducted at Queen Elizabeth Central Hospital (QECH), Blantyre, Malawi. In 2010 HIV prevalence in pregnant women in this region was 18% and overall seroprevalence in Malawian children less than 14 years old was estimated to be 2.7%.^12^ Malaria transmission in rural communities around Blantyre occurs year-round peaking during the rainy season (November–June).

Children diagnosed with HIV were followed up in paediatric HIV clinics, received daily preventive co-trimoxazole and, from 2001 and when eligible, combination antiretroviral therapy ([ART] lamivudine, stavudine and nevirapine; Triomune, Cipla). Routine CD4 quantification and WHO staging were introduced in 2006.

### Patients

As part of a longstanding clinico-pathological study of CM in Blantyre,^13^ Malawian children aged 6-months to 12-years presenting to QECH with clinical CM were recruited and managed on a paediatric research facility during consecutive rainy seasons from February, 1996 to June, 2011.

### Management

Patients with CM were treated with intravenous quinine for at least 24 h and then switched to oral drugs (Sulphadoxine-pyrimethamine pre-2007 or Lumefantrine-artemether). Ward rounds by experienced clinicians were conducted twice daily.

From 2001 all patients whose HIV status was unknown were tested for HIV after a parent or legal guardian gave consent. Prior to 2001 HIV tests were conducted retrospectively on stored samples. In fatal cases where HIV-status was unknown, it was done posthumously. Ethical approval was obtained for this retrospective and posthumous testing.

### Blood collection and diagnostic tests

Venous blood was collected on admission. Plasma was stored at −80 °C for ELISA tests. A full blood count (Coulter Counter, Becton–Dickinson, New Jersey), blood culture (BACTEC 9120, Becton–Dickinson) and thick and thin blood smears (Field staining) were performed on all patients. Peripheral parasite density was calculated using the patients' individual full blood count. HIV testing was performed with two rapid tests, Determine (Abbott laboratories, Green Oaks, IL) and Unigold (Trinity Biotech PLC, Bray, Ireland). A third test was used to resolve discrepancies (Capillus, Trinity Biotech). For patients <18-months HIV status was determined by PCR (Amplicore, Roche, Pleasanton, CA). Unless contraindicated, a lumbar puncture was performed. Patients with visibly cloudy CSF were excluded from the analysis.

### ELISA tests

We determined HRP2 levels from stored plasma of a subset of patients, including all patients admitted in 2009, for a previous study.^14^ For patients admitted in 2010 and 2011, TNF, IL10 and sICAM-1 were determined from stored plasma using commercial ELISA kits: (R&D, Minneapolis; DY210, DY217B and DY720).

### Statistical analysis

Analysis was performed using Stata software (Version 10.0-Statacorps, Texas USA). Non-normally distributed continuous variables were compared using a Mann–Whitney U test and summarized using medians and interquartile ranges. Associations between categorical variables were assessed using the Fisher's Exact test. The Cox proportional hazards model was used to analyse time to death by HIV status and hazard ratios with 95% confidence intervals (CI) reported. The relationship between mortality and baseline variables was assessed using odds ratio (OR). The baseline variables of interest in this assessment were lactate levels, gender, HIV status and age. A logistic regression model was fitted to obtain unadjusted and adjusted OR in this assessment for the four baseline variables. The OR, associated 95% confidence intervals and the p-values have been reported. Graphical summaries have also been presented for the variables of interest. These include Kaplan Meier plots for the time to event data, histograms and dot plots for summarising continuous data between groups. All tests were considered statistically significant at 5% significance level.

### Ethics statement

The study was approved by ethics committees of the College of Medicine, University of Malawi (P.02/10/860), Michigan State University, USA and Liverpool School of Tropical Medicine, UK (09.74), of note specific ethical approval was obtained for retrospective and posthumous HIV-testing.

## Results

### Retrospective review by HIV status of all retinopathy-positive cases prospectively recruited into the Blantyre clinico-pathological study of CM

**Figure 1:**
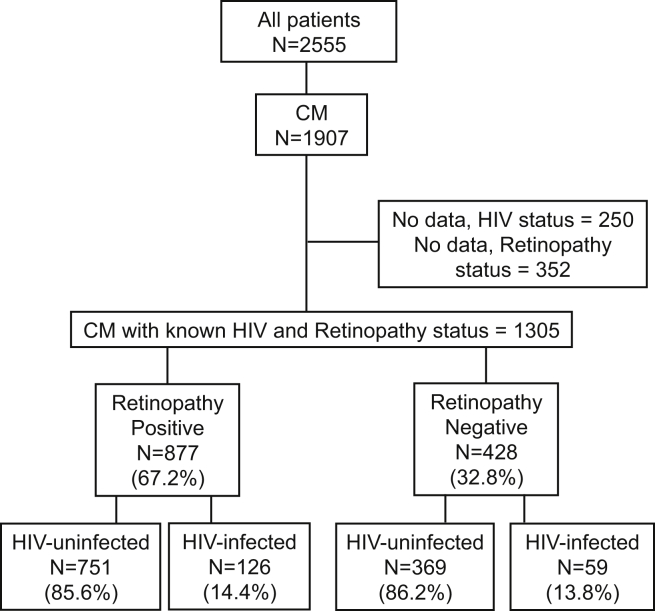
**Study profile of the children included**.

Of 2555 children admitted to the paediatric research ward from 1996 to 2011, 1905 children fulfilled the WHO definition of CM. Of these 1657 (86.9%) had data on HIV status. 877 children had malarial retinopathy on fundoscopy and 126 (14.4%) of these retinopathy positive children were HIV-infected (Fig. 1). Data on WHO HIV staging and CD4 counts of patients prior to 2007 were unavailable. Of the 37 HIV-infected retinopathy positive CM cases admitted after 2007, WHO staging was available for 19; 10 were WHO stage I, 1 was stage II, 4 in stage III and 4 stage IV. Two children were on ART at the time of admission.

**Figure 2:**
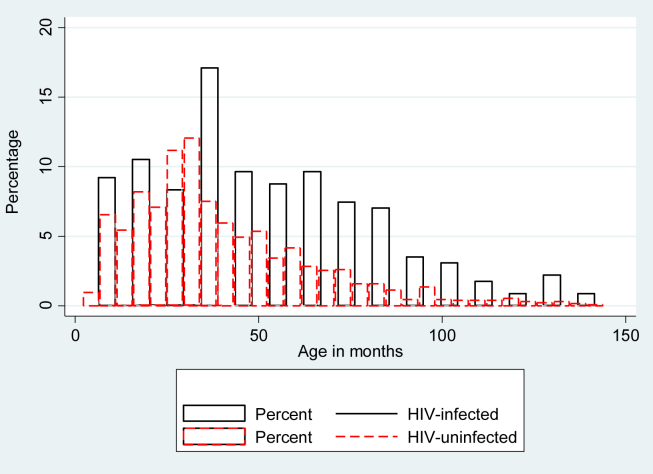
**Age distribution of children by HIV status**. Red represents HIV negative and black HIV-positive. The heights of the bars give the proportion of CM in each age group. HIV-infected children were older with median age of 48 months, IQR 32–72; HIV negative 33 months IQR 23–50; P < .001.

As shown in Fig. 2, compared with HIV-uninfected children HIV-infected children with retinopathy positive CM were older (HIV-infected 49.5 months, IQR 32.0–72.0; HIV-uninfected patients 34 months, IQR 24.0–52.0, p < 0.001). HIV-infected children were also more likely to have evidence of co-morbidities, specifically abnormal chest auscultation findings (crackles, wheeze: HIV-infected 13.9%, HIV-uninfected 7.1%; p = 0.018; Table 2) and poor nutritional status: lower weight-for-age z-scores (HIV-infected median −2.08, IQR −2.82 to −1.25; HIV-uninfected −1.61, IQR-2.37 to −0.86, p = 0.001) and lower median mid upper arm circumference-for-age z-score, (HIV-infected −1.29, IQR −2.07 to −0.67; uninfected −1.06, IQR −1.67 to −0.37; p = 0.014).

Other key symptomatic features of illness prior to presentation to hospital including vital observations (respiratory rate, pulse, blood pressure) and the duration of presenting features (fever, convulsions, coma) were similar between HIV-infected and uninfected children (Table 2). The number of children who had received either oral or parenteral antimalarial treatment (chloroquine, sulphadoxine pyrimethamine, lumefantrine-artemether or quinine) before arrival at QECH was also not affected by HIV status (HIV-infected, 29.7%, HIV-uninfected, 29.0%, p = 0.91). Characteristics of retinopathy negative cases are discussed below.

### Baseline laboratory findings on admission

**Figure 3:**
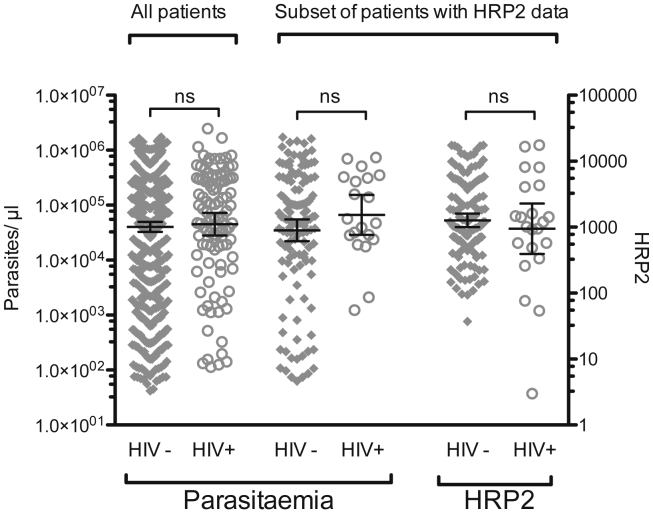
**Similar peripheral parasite density and HRP2 levels in HIV-infected HIV-uninfected children with retinopathy positive CM**. Horizontal lines represent medians and bars represent interquartile range. There was no difference in either: (A) peripheral parasitaemia or (B) HRP2 levels between the two groups, p values 0.88 and 0.78, respectively. Peripheral parasite density in the subset of HIV-positive and HIV-negative patients who had HRP2 measured was similar to parasite density in their respective groups in the study overall.

**Figure 4:**
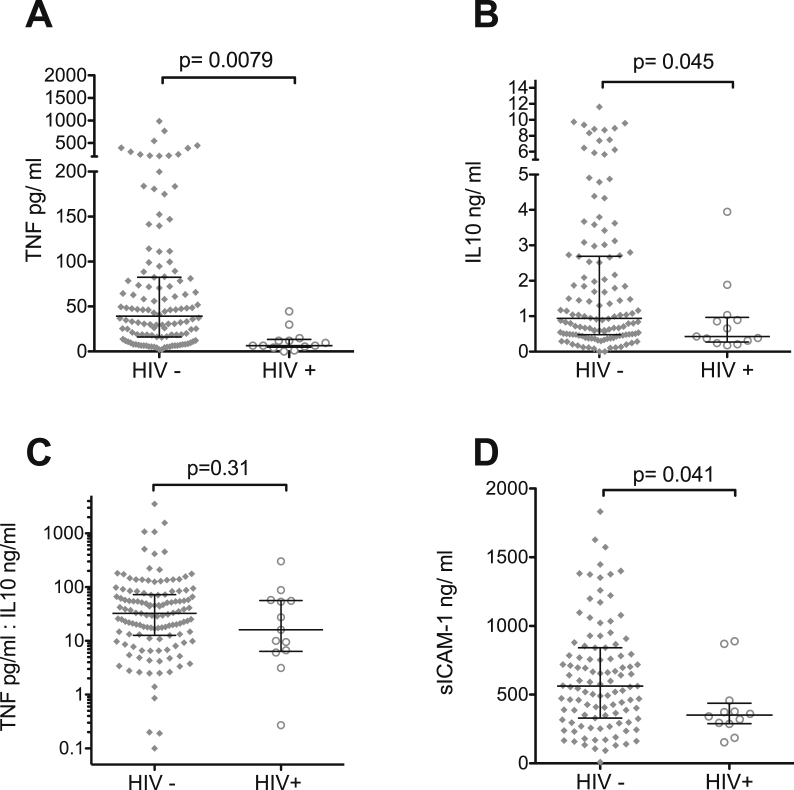
**Soluble plasma markers in HIV-infected and HIV-uninfected children with CM**. Median admission plasma levels measured by ELISA of TNF, ICAM-1 and IL10 in HIV-positive children were significantly lower than those of HIV negative children. (A, B, C) however the ratio of TNF to IL10 (D) was similar in the two groups. Horizontal lines represent medians and bars represent interquartile range.

Geometric mean peripheral parasite densities were similar between HIV-infected (45,059 parasites/μl, 95% CI 28,098–72,258) and HIV-uninfected children (40,195 parasites/μl, 95% CI 32,771–49,301 p = 0.68, Table 3), as were geometric mean HRP2 concentrations between the subset of 139 HIV-uninfected (1268 ng/ml, 95% CI 1002–1604) and 24 HIV-infected children (946 ng/ml, 95% CI 393–2279; p = 0.39; Table 3 and Fig. 3) in whom HRP2 levels were measured. The geometric mean and 95% CI for peripheral parasite density of this subset in whom HRP2 was measured was similar to the overall cohort, suggesting it was representative of the cohort as a whole (Fig. 4). Other laboratory parameters were similar between HIV-infected and HIV-uninfected children (Table 3). Laboratory characteristics of retinopathy negative cases are discussed below.

### Disease progression and outcome

It took HIV-infected children with retinopathy positive CM longer to fully recover consciousness (coma resolution time: HIV-infected, median 42.0 h, IQR 22.0–70.0; HIV-uninfected children, median 34.0 h, IQR 18.0–56.0; p = 0.0085, Supplemental Table 1). However parasite clearance time, (the time in hours from admission until two consecutive thick smears were negative) was similar in HIV-infected (42.0 h, IQR 30.0–54.0 h) and uninfected children (42 h, IQR 28.0–54.0 h; p = 0.628) as was time to fever clearance (the time in hours from admission until the last recorded temperature >37.5 °C; IQR 20.0–74.0 and 20.0–54.0; p = 0.0689).

The unadjusted hazard ratio for fatal outcome was not significant (HIV-infected 24.8%, HIV-uninfected 18.5%; Hazard ratio 1.104; 95% CI 0.645–1.887; p = 0.13). Among retinopathy positive children this remained non-significant when analysis was restricted to children <5 years old (27.2% and 18.1%; p = 0.07) or children <3 years old (25% and 12.8%; p = 0.08). There was also no difference in survival profile between the HIV-infected and uninfected children (p = 0.720, Supplemental Fig. 1).

In view of significant changes to the management of HIV and malaria (e.g. the roll out of ART for HIV in 2007 and lumefantrine-artemether for uncomplicated malaria in 2012) and progression of the HIV epidemic in Malawi,^12^, ^15^ we analysed the data in five-year periods (1996–2000; 2001–2005; 2006–2011), looking at patient characteristics, clinical course and mortality (Supplementary Table 2). Each period gave similar results to the overall cohort.

Within a logistic regression model adjusting for age (in months) and sex, among the retinopathy positive children, lactate was the only independent predictor of mortality (OR 1.10 per mmol increase, 95% CI 1.04–1.16, p < 0.001; Supplemental Tables 2 and 3).

### Retinopathy negative cases

In the retrospective review of patients admitted 1996–2011, 428 children were retinopathy negative and of these 59 children (13.8%) were HIV-infected (Fig. 2). As with retinopathy positive cases HIV-infected retinopathy negative cases were also older than HIV-uninfected cases (Table 2). However in contrast to retinopathy positive cases, HIV-infected children had differences compared to HIV-uninfected children in a number of laboratory indices. HIV-infected retinopathy negative children had a higher parasitaemia geometric mean parasite density (74,416 parasites/μl; 95% CI 49,648–111,541) than HIV-uninfected children (34,191 parasites/μl; 95% CI 27,137–43,078, p = 0.010, Table 3). Geometric mean HRP2 level was also higher in HIV-infected children (214 ng/ml, 95% CI 99.5–459) compared with HIV-uninfected children (86.3 ng/ml, 95% CI 58.3–128), although not statistically significant (p = 0.19). Consistent with previous data^14^ HRP2 levels were significantly lower in retinopathy negative than in retinopathy positive CM cases (p < 0.001). Median haematocrit (HIV-infected 27%, IQR 20–30%; HIV-uninfected 29%, IQR 25–33%; p = 0.0014) and median platelet levels (HIV-infected 78 × 10^9^/L, IQR 34–178 × 10^9^/L; HIV-uninfected 148 × 10^9^/L, IQR 61–225 × 10^9^/L; p = 0.0047) were significantly lower in HIV-infected children. Other clinical features were not significantly different between HIV-infected and uninfected retinopathy negative children (Table 2) and there was not a significant effect of HIV- status on mortality (HIV-infected 13.8%, HIV-uninfected 9.81%; Hazard ratio 1.47; 95% CI 0.55–3.46; p = 0.36). Taken together, comparison of the effect of HIV on CM cases by retinal status indicates that HIV status has a larger effect on clinical features among retinopathy negative cases than among retinopathy positive cases.

### Plasma cytokine levels in HIV-infected and uninfected retinopathy positive CM patients

In total, 224 children with a clinical diagnosis of CM were admitted in 2010 and 2011. Of these, 153 children had retinopathy positive CM and had data available on HIV status: 137 were HIV-uninfected and 15 HIV-infected. Plasma for cytokine analyses was missing or insufficient in 18 cases: 15 HIV-uninfected and 3 HIV-infected.

TNF levels on admission in the 12 HIV-infected children (median 6.47 pg/mL; IQR 4.92 pg/mL to 13.4 pg/mL) were markedly lower than in the 122 HIV-uninfected children (median 39.3 pg/mL; IQR 16.1 pg/mL to 82.6 pg/mL; p = 0.0079; Fig. 4A). IL10 levels were also significantly lower in the HIV-infected (median 0.43 ng/mL; IQR 0.27 ng/mL to 0.96 ng/mL) compared to HIV-uninfected children (median 1.01 ng/mL; IQR 0.49 ng/mL to 2.96 ng/mL; p = 0.045; Fig. 4B). The TNF-to-IL10 ratio was not significantly affected by HIV status (HIV-uninfected, median 33.4; IQR 13.3 to 74.1; HIV-infected, median 16.1; IQR 6.39 to 56.5; Fig. 4C).

Sufficient plasma was available to measure sICAM-1 for 107 HIV-uninfected and 12 HIV-infected retinopathy positive children. sICAM-1 levels were significantly lower in the HIV-infected (median 350 ng/mL; IQR 289–437 ng/mL) than in the HIV-uninfected children (median 563 ng/mL; IQR 330–841 ng/mL; p = 0.04; Fig. 4D).

## Discussion

We have used a large cohort of well-characterized patients and a stringent definition of CM to explore the effect of HIV on CM in an attempt to unravel the controversy relating to the role of systemic inflammation in CM pathogenesis. HIV had a marked effect on the inflammatory response to CM: HIV-uninfected children with CM had substantially raised TNF and ICAM-1 levels compared to HIV-infected CM children. IL-10 levels were also lower in HIV-infected children but the TNF-to-IL-10 ratio remained similar, hence there was not a clear pro/anti-inflammatory cytokine imbalance. Despite this marked blunting of the inflammatory response, HIV-infected and uninfected children with retinopathy positive CM had a similar outcome.

It is likely that the lack of systemic inflammatory response in HIV-positive children is at least in part due to impaired CD4 T-cell function. Peripheral blood mononuclear cells from HIV-infected adults have decreased TNF production (T-helper 1 cytokine) in response to challenge with *P. falciparum in vitro*.^11^ Therefore through abrogating the cytokine response to malaria infection in HIV-infected individuals, HIV has provided a ‘natural experiment’, shedding light on the role of the systemic cytokine response in CM pathogenesis. With regards to the pro-inflammatory T-helper 1 response, it has been long postulated that pro-inflammatory cytokines, particularly TNF, may provide a double-edged sword in malaria outcome. On the one hand, TNF may play a critical role in the immune control of overall parasite burden which may be an important determinant of disease severity and outcome.^16^ On the other hand, high levels of TNF and a cytokine storm have been postulated to be critical in the development and outcome of severe and CM.^16^, ^17^ Here we show that HIV-infected children have retinopathy positive CM with similar clinical features, peripheral parasite density, HRP2 levels and outcome, despite a markedly blunted cytokine response, to HIV-uninfected children with retinopathy-positive CM. These findings imply that substantially raised systemic TNF levels and a cytokine storm are not necessary for the development of CM. Hochman et al. found a higher level of platelet and monocyte accumulation in histologic sections of cerebral vessels in HIV-infected cases in association with sites of iRBC sequestration compared to HIV-uninfected cases.^18^ Given the localised nature of these pathologies and given our data indicating a lack of significant systemic inflammation in HIV-infected children, these histopathological findings suggest that specific interactions between iRBC and either the endothelium itself or other host cells in close proximity may be important in disease pathogenesis.

Examining the genes expressed by iRBC sequestered in the brain, Tembo et al. demonstrated that different var genes were expressed between HIV-infected and HIV-uninfected children.^19^ Var genes control the surface proteins expressed on iRBC and thereby the host endothelial receptors with which they bind and interact. Taken together these findings indicate that the local histological differences observed by Hochman and colleagues between HIV-infected and uninfected children may reflect differences in the nature of the iRBC-endothelial interaction. What factors lead to different var gene expression in HIV and how this affects the iRBC-host cell interaction remains to be determined but elucidating this may shed further light on CM pathogenesis in both HIV-infected and uninfected children.

The lack of a significant difference in mortality rate between HIV-infected and uninfected CM cases here seem to contradict a recent publication that found a higher mortality in HIV-infected children admitted to our facility.^18^ The principal difference between the analyses is that the earlier publication used a purely clinical definition of CM whereas we used retinopathy status to improve specificity and hence only include cases for which coma is more likely to be caused by malaria.^18^ By including all cases, whether true retinopathy positive CM or not, the earlier study had a slightly larger sample size and we cannot exclude that this may have increased the statistical power to detect a significant mortality difference. However it is also possible that the mortality difference associated with HIV in the earlier analysis is unrelated to any effect of HIV on CM but instead due to confounding factors – in particular that HIV is highly associated with death from other comorbidities, such as bacterial infection. The effect of such confounders is likely to be stronger in an analysis that, due to lack of diagnostic specificity, includes a significant proportion of children whose coma is not caused by malaria. Different specificities of the clinical case definitions and different rates of co-morbidities may also be important in explaining different and apparently inconsistent effects of HIV on mortality reported in previous severe malaria studies.

Although our data are derived from a large cohort of CM patients, our study was limited by the relatively small number and small volume of plasma samples available. We were therefore only able to measure one Th1 and one Th2 cytokine on presentation to hospital. Serial blood samples may have provided a more complete picture. We also did not undertake long-term follow up to reliably determine whether HIV affects the risk of subtle or slowly developing neurodevelopmental sequelae following CM.

In conclusion, when CM is defined precisely in African children, HIV has a marked impact on the cytokine response but little effect on either parasite density or the clinical course of CM. Taken with other recent studies these data point towards local iRBC-associated effects rather than systemic inflammation as the primary driver of pathogenesis in human CM.

## Funding

This work was supported by grants from the NIH (T.E.T., 5R01AI034969-14) and a Clinical Fellowship from The Wellcome Trust, United Kingdom (C.A.M, 88758). The Malawi-Liverpool-Wellcome Clinical Research Programme is supported by core funding from The Wellcome Trust, UK.

## Contributions

Emmie W. Mbale, Christopher A Moxon, Macpherson Mallewa, Terrie Taylor & Robert Heyderman had the idea for the study. Terrie Taylor, Malcolm E Molyneux and Karl Seydel coordinated the overarching malaria pathogenesis study. Ngawina Chisala, Karl Seydel and Christopher A Moxon performed the ELISA experiments. Simon Glover conducted fundoscopic examinations. Maganizo Chagomerana did the data entry and management of the database. Mavuto Mukaka, Christopher Moxon and Emmie Mbale performed statistical analysis. Sofia Omar assisted in data collection. Emmie Mbale and Christopher Moxon wrote the first draft of the report with input from other authors.

## Conflict of interest

The authors have no conflicts of interest to declare.

Some of the data included in this manuscript was presented at American Society of Tropical Medicine and Hygiene Annual meeting December 2012.

## Figures and Tables

**Table 1:** Summary of publications providing data on the interaction between HIV and cerebral malaria (CM).

First author and year of publication	Participants	Study aim	Number of cases with coma	Number of cases with HIV	Number of cases with HIV & coma	Retinal examination used to refine CM diagnosis?	Key findings	Findings specific to HIV and cerebral malaria
Bronzan at al, 2007	1388 hospitalised Malawian children with severe falciparum malaria	To examine the association between HIV, bacteraemia and outcome.	1217	179 (16%)	150 (86 coma only; 64 anaemia and coma)	No	Association between non-typhoidal salmonella bacteraemia, HIV infection and malaria; 2) HIV+ children were older than HIV− children	1) HIV+ children more likely to have anaemia than coma as a complication 2) Coma occurs in older children than anaemia.
Cohen et al., 2005	336 hospitalised South African (Soweto) adults with falciparum malaria	To assess the effect of HIV infection and CD4 count on the risk of developing severe malaria	7	110 (33%)	3	No	HIV infection was associated with a higher risk of severe malaria and death. Risk compounded by low CD4 count.	Insufficient numbers of comatose children.
Grimwade et al., 2004	613 South African (KwaZulu-Natal) adults with falciparum malaria	To measure the association between HIV status and outcome from malarial infection	32	180 (29.9%)	16	No	1) Increased risk of severe disease in HIV+ adults compared to HIV−; 2) Increased mortality in HIV+ cases: 3) Parasitaemia not affected by HIV status.	Higher proportion of HIV+ cases with coma. Other parameters not examined separately in comatose and non-comatose adults (as not objective of study).
Grimwade et al., 2003	663 South African (KwaZulu-Natal) children with falciparum malaria	To describe associations among HIV status, presentation and outcome from malaria in children	11	67 (10.1%)	3	No	HIV infection associated with severe/complicated malaria	Trend toward higher proportion of HIV+ cases with coma.
Hendricksen et al., 2012	655 Hospitalised children (68 adults) with falciparum malaria	To determine the impact of HIV on clinical signs, complications, and disease outcome of severe malaria	495 children; 48 adults	74 children (14.9%); 49 adults (72.1%)	54 children; 35 adults	No	1) Higher mortality and parasite burden in HIV+ children 2) Higher proportion of HIV+ children with respiratory distress/acidosis 3) Higher rate of clinical comorbidities (e.g. pneumonia; 'clinical' sepsis) in HIV+ children.	Proportion of children with coma not affected by HIV status. Other parameters not examined separately in comatose and non-comatose children (as not objective of study).
Niyongabo et al., 1994	31 Burundian adults with cerebral malaria	To identify risk factors for poor prognostic in cerebral malaria in adults	31	12 (38.7%)	12	No	No clear effect of HIV-1 infection on presenting features of cerebral malaria or outcome.	As per key findings but low numbers of patients.
Imani et al., 2011	100 Ugandan children with CM compared with 132 uncomplicated malaria and 120 aparasitemic controls	To determine whether there is an association between HIV and the development of CM	100	15 overall (4.3%)	9 (9%)	No	Higher proportion of children with CM are HIV+ compared with children with uncomplicated malaria or no malaria	As per key findings. No specific information on subgroup of comatose HIV+ children (as not objective of study)
Hochmann et al., 2015	Post-mortem study of 30 children 15 of whom were HIV positive. Retrospective review of children with a clinical diagnosis of CM.	1. To examine the effect of HIV on histological features post-mortem. 2. To examine the effect of HIV on mortality.	2009	232 (11.5%)	232 (11.5%)	Yes for post-mortem cases No for mortality analysis	1. Greater platelet, monocyte and neutrophils in HIV positive 2. Higher mortality in comatose HIV-infected than uninfected children	As per key findings.

**Table 2:** Clinical Characteristics of retinopathy positive and retinopathy negative children included in the study, compared by HIV status.

Variable	Retinopathy positive					Retinopathy negative				
HIV-uninfected (N = 751)		HIV-infected (N = 126)		p	HIV-uninfected (N = 428)		HIV-infected (N = 59)		p
N	Value	N	Value	N	Value	N	Value
Male sex, N (%)	749	47.53%	126	52.38%	0.336	368	50.32%	59	57.14%	0.8056
Age, months (IQR)	751	34 (24–52)	126	49.5 (32–72)	<0.0001	369	36 (24–57)	59	49 (26–72)	0.0157
Weight-for-age, z-score (IQR)	749	−1.61 (−2.37, −0.86)	126	−2.08 (−2.82, −1.25)	0.0001	351	−1.77 (−2.59 to −1.00)	59	−1.85 (−2.66 to −0.97)	0.494
MUAC-for-age, z-score (IQR)	600	−1.06 (−1.70, −0.44)	74	−1.41 (−2.28, −0.72)	0.014	274	−1.22 (−1.88 to −0.42)	30	−1.37 (−2.40 to −0.20)	0.437
Fever duration at admission, hrs (IQR)	728	48 (36–72)	122	48 (24–72)	0.7858	297	48 (24–72)	59	37 (16–72)	0.89
Duration of coma at admission, hrs (IQR)	624	6 (3–12)	104	6 (3.5–15)	0.8891	369	5 (3–9)	59	4 (2–7)	0.68
History of convulsions, N (%)	671	482 (71.83%)	119	78 (65.55%)	0.189	296	85.67%	48	79.59%	0.811
History of antimalarials (%)^a^	679	197 (29.01%)	118	35 (29.66%)	0.913	369	110 (29.81%)	59	17 (28.81%)	0.876
Temp, °C (IQR)	751	38.7 (37.9–39.5)	126	38.7 (38.0–39.8)	0.1533	369	38.7 (37.6–39.6)	49	38.9 (37.7–39.8)	0.5302
Pulse rate, per min (IQR)	750	154 (140–171)	126	160 (136–171)	0.6597	369	152 (134–168)	49	148 (134–160)	0.1126
Respiratory rate, per min (IQR)	750	44 (38–54)	126	48 (36–58)	0.3081	369	42 (36–54)	49	44 (32–56)	0.8453
Systolic BP, mmHg (IQR)	729	100 (91–110)	122	100 (90–114)	0.8616	369	104 (95–120)	49	104 (90–119)	0.4854
Abnormal chest findings, N (%)	746	53 (7.10%)	122	17 (13.93%)	0.018	253	6.72%	23	9.30%	0.72
Lymphadenopathy, N (%)	327	12 (3.67%)	46	4 (8.7%)	0.121	*102*	*6.86*%	*11*	*27.27*%	0.057
Jaundice, N (%)	325	26 (8.00%)	46	4 (8.70%)	0.456	*102*	*1.96*%	*4*	*9.09*%	0.267

N = Number, IQR = Interquartile range, MUAC = Mid upper arm circumference, WBC = White blood cell count, BP = blood pressure, min = minute, hrs = hours.

a Antimalarials taken included Chloroquine, SP, quinine, LA.

**Table 3:** Laboratory characteristics of retinopathy positive and retinopathy negative children, compared by HIV status.

Variable	Retinopathy positive					Retinopathy negative				
HIV-uninfected (N = 751)		HIV-infected (N = 126)		p	HIV-uninfected (N = 428)		HIV-infected (N = 59)		p
N		N		N		N	
Parasite density, per μl – geometric mean (95% CI)	735	40,195 (32,771–49,301)	117	45,059 (28,098–72,258)	0.802	360	34,191 (27,137–43,078)	56	74,416 (49,648–111,541)	0.001
HRP2, ng/mL – geometric mean (95% CI)	139	1268 (1002–1604)	24	946 (393–2279)	0.965	114	86.3 (58.3–128)	10	214 (99.5–459)	0.390
Haematocrit, % – median (IQR)	747	19.5 (15.0–24.7)	125	18 (14–24)	0.179	369	29 (25–33)	59	27 (20–30)	0.001
Blood WBC, ×10^9^/L – median (IQR)	700	7100 (10,200–14,900)	113	10,700 (7500–16,100)	0.434	369	10.0 (6.9–14.0)	45	9.3 (6.5–13.2)	0.074
Platelet count, ×10^9^/L – median (IQR)	669	57,000 (34,000–88,000)	106	64,500 (41,000–98,000)	0.078	323	148 (61–225)	53	78 (34–178)	0.005
aPlasma Lactate, mmol/L – median (IQR)	440	6.9 (3.60–11.30)	74	7.85 (4.34–12.7)	0.150	162	5.3 (3.1–8.3)	16	7.25 (4.6–13.0)	0.088
Blood Glucose, mmol/L – median (IQR)	745	5.7 (4.31–7.30)	124	6.00 (4.30–7.85)	0.348	368	6.2 (3.7–7.8)	59	5.8 (3.7–7.8)	0.123
CSF Pleocytosis,^b^ number (%)	751	3.18%	126	5.47%	0.195	264	6.06%	45	11.11%	0.307
Bacteraemia, number (%)	734	4.36%	125	8.00%	0.110	362	2.76%	56	3.57%	0.668

HRP2 = *P. falciparum* Histidine rich protein 2.

a Plasma lactate testing was only available from 2001.

b CSF pleocytosis defined as >10 × 10^9^ WBC per mL.

## Appendix A Supplementary data

Supplemental Figure 1 **Kaplan–Meier curve showing similar mortality outcome in HIV-infected and HIV-uninfected children (Time in days, 0 is the time of admission)**: Survival profile was not significantly different between HIV-infected and uninfected children. Hazard ratio 1.10, 95% confidence interval 0.65–1.89, p = 0.72.
