# Supplementary material for: HIV coinfection influences the inflammatory response but not the outcome of cerebral malaria in Malawian children
Source: J Infect. 2016 Sep;73(3):189–99. doi: 10.1016/j.jinf.2016.05.012 (PMC4990000; doi:10.1016/j.jinf.2016.05.012)
Supplement: Supplemental Figure 1 — Kaplan–Meier curve showing similar mortality outcome in HIV-infected and HIV-uninfected children (Time in days, 0 is the time of admission): Survival profile was not significantly different between HIV-infected and uninfected children. Hazard ratio 1.10, 95% confidence interval 0.65–1.89, p = 0.72. [file mmc1.docx]

| Variable | RETINOPATHY POSITIVE | | | | | RETINOPATHY NEGATIVE | | | | |
| --- | --- | --- | --- | --- | --- | --- | --- | --- | --- | --- |
|  | **HIV-uninfected (N=751)** | | **HIV infected (N=126)** | |  | **HIV-uninfected (N=369)** | | **HIV-infected (N=59)** | |  |
|  | N | Value | N | Value | p | N | Value | N | Value | p |
| Parasite Clearance Time, hours (IQR) | 596 | 42 (30-54) | 97 | 42 (28-54) | 0.63 | 369 | 36 (24 - 48) | 59 | 42 (24 - 54) | 0.37 |
| Fever Clearance Time, hours (IQR) | 591 | 32 (20-54) | 90 | 37 (20-74) | 0.069 | 369 | 24 (6 - 38) | 59 | 32 (6 - 58) | 0.10 |
| Coma Resolution Time, hours (IQR) | 563 | 34 (18-56) | 90 | 42 (22-70) | 0.0085 | 369 | 18 (10 - 38) | 59 | 16 (6 - 32) | 0.32 |
| Fatal-outcome, number (%) | 750 | 140 (18.7%) | 126 | 30 (23.8%) | 0.27 | 367 | 36 (9.81%) | 58 | 8 (13.8%) | 0.86 |
| Survived with sequelae, number (%) | 750 | 64 (8.53%) | 126 | 9 (7.14%) | 0.51 | 367 | 38 (10.4%) | 58 | 6 (10.3%) | 0.56 |

Supplemental table 1. Disease progression and outcome in retinopathy positive and retinopathy negative children, compared by HIV status.

Sequelae refers to neurological sequelae including focal muscle weakness, visual or hearing defects, gross cognitive deficit or epilepsy.

Supplementary table 2: Comparison between year categories: 1996-2000; 2001-2005; 2006-2011

| **Year category** | **1996-2000;** | | | **2001-2005** | | | **2006-2011** | | |
| --- | --- | --- | --- | --- | --- | --- | --- | --- | --- |
| **N ret positive CM** | 310 | | | 143 | | | 424 | | |
| **HIV prevalence** | 36/310 (11.61%) | | | 34/143 (23.78%) | | | 56/424 (13.21%) | | |
|  | HIV neg | HIV pos | P value | HIV neg | HIV pos | P value | HIV neg | HIV pos | P value |
| **Male sex** | 128/273 46.89% | 16/36 (44.4%) | 0.860 | 14/34 (41.18%) | 50/108 (46.30%) | 0.694 | 178/368 (48.37%) | 36/56 (64.27%) | 0.031 |
| **Age (months)** | 32 (21-50) | 45.5 (19-43) | 0.0039 | 29 (19-43) | 50.5 (34-75) | 0.0025 | 37 (28-54.5) | 50.5 (34-75) | 0.0012 |
| **Fever duration (hours)** | 48 (36-72) | 48 (24-72) | 0.7591 | 48 (24-72) | 48 (26-72) | 0.8851 | 48 (48-72) | 62.5 (25-72) | 0.7100 |
| **Coma duration (hours)** | 5 (3-11) | 6 (3-12) | 0.8834 | 6 (3-10) | 6 (4-12) | 0.6650 | 8 (4-15) | 7 (4-22) | 0.8184 |
| **Convulsions duration (hours)** | 4 (2-11) | 4 (2-8) | 0.8918 | 6 (3-10) | 4.5 (2.5--9.5) | 0.4628 | 8 (4-14) | 7.5 (4-15) | 0.9346 |
| **Temp on admission (^0^C)** | 38.6 (37.7-39.4) | 38.5 (38-39.7) | 0.7970 | 38.7 (38-39.7) | 38.9 (38.2-39.9) | 0.4090 | 38.8 (38-39.5) | 38.8 (38.1-40.0) | 0.2485 |
| **Heart rate on admission (beats/min)** | 152 (138-168) | 152 (138-168) | 0.5128 | 156 (140-168) | 160(126-168) | 0.6598 | 155 (140-174) | 159 (140-175) | 0.6342 |
| **Respiratory rate on admission (cycles/min)** | 44 (36-56) | 48 (32- 55) | 0.9439 | 48 (40-59) | 36 (48-60) | 0.9697 | 44 (40-54) | 48 (41-56) | 0.1516 |
| **Systolic BP on admission (mmHG)** | 110 (98- 120) | 110 (100- 120) | 0.5712 | 110 (100- 120) | 90 (105-120) | 0.8878 | 88 (96- 105) | 88 (96-103) | 0.7895 |
| **Transfusion given Y/N** | 21/137 (15.33%) | 1/24 (4.17%) | 0.203 | 5/64 (7.81%) | 3/16 (18.75%) | 0.194 | 179/296 (60.47%) | 31/48 (64.48%) | 0.635 |
| **Neck stiffness** | 16/265 (6.04%) | 3/36 (8.33%) | 0.484 | 4/107 (3.74%) | 1/32 (3.03%) | 1.000 | 10/357 (2.72%) | 4/56 (7.14%) | 0.100 |
| **Jaundice noted** | N/A | N/A | N/A | N/A | N/A | N/A | 26/325 (8%) | 4/46 (8.7%) | 0.777 |
| **Laboratory results** |  |  |  |  |  |  |  |  |  |
| **Admission parasitaemia (per ul)** | 104168.50 (8427.00-339544.50) | 109528 (5042.5- 323125) | 0.7527 | 72701.50 (12480- 321893) | 52560 (18332.50- 227715.50) | 0.6301 | 69840 (11880- 288000) | 72960 (17840-311952) | 0.7381 |
| **Admission haematocrit (%)** | 20 (15- 26) | 18 (14-25) | 0.2165 | 18 (15-24) | 20.50 (15- 24) | 0.9564 | 19 (16-23.20) | 18 (13.90-24) | 0.3941 |
| **Admission blood white cell count (x10^9^/l)** | 11000 (7400-16800) | 10700 (6800- 14600) | 0.4997 | 10700 (7,500-15,100) | 10100 (6600- 17300) | 0.9133 | 9600 (6800- 14100) | 11100 (8100- 18800) | 0.1231 |
| **Platelet count (x10^9^/l)** | 60000 (38000-87000) | 68000 (45000- 89000) | 0.5140 | 64000 (38000-108000) | 93000 (57000-141000) | 0.1082 | 54000 (32000-86000) | 56000 (31000- 95000) | 0.4443 |
| **Admission lactate (mmol/l)** | N/A | N/A | N/A | 8.75 (4.70-13.60) | 7.10 (4.40-14.20) | 0.9076 | 6.40 (3.30-10.90) | 8.55 (3.60-12.65) | 0.1592 |
| **Admission glucose (mmol/l)** | 5.40 (4-6.90) | 5.10 (3.80-6.10) | 0.4161 | 5.60 (4.50-7.80) | 6.80 (5.30- 9.00) | 0.0560 | 6 (4.45-7.60) | 6.20 (4.65-7.60) | 0.8081 |
| **Disease progression and outcome data** |  |  |  |  |  |  |  |  |  |
| **Length of stay (days)** | 3 (2-4) | 3 (2-4) | 0.6260 | 3 (2-4) | 4 (3-6) | 0.0059 | 3 (3-4) | 3.50 (1.50-5) | 0.6039 |
| **Parasite clearance time (hours)** | 42 (30-53) | 36 (21-51) | 0.2158 | 36 (24-48) | 45 (30-54) | 0.2192 | 42 (30-54) | 48 (30-54) | 0.2499 |
| **Fever clearance time (hours)** | 32 (18-48) | 24 (12-48) | 0.5720 | 28 (16-38) | 31 (26-87) | 0.0243 | 36 (20-58) | 42 (20-78) | 0.0750 |
| **Coma resolution time (hours)** | 28 (15.5-54) | 35.5 (22-60) | 0.1082 | 30 (15-47) | 45 (32-70) | 0.0202 | 38 (20-58) | 45 (21-72) | 0.2592 |
| **Mortality** | 49/274 (17.88%) | 8/36 (22.22%) | 0.499 | 30/109 (27.52%) | 7/34 (20.59%) | 0.505 | 61/367 (16.62%) | 14/56 (25%) | 0.134 |
| **Neurological sequelae** | 26/274 (9.49%) | 1/36 (2.78%) | 0.383 | 10/109 (9.17%) | 4/34 (11.76%) | 0.658 | 28/367 (7.63%) | 4/56 (7.14%) | 0.308 |

**Supplemental table 3: Univariate logistic analysis for lactate, gender, age and HIV status**

| Variable | OR | 95% Confidence interval | p-value |
| --- | --- | --- | --- |
| Age | 0.998 | 0.991 1.005 | 0.52 |
| Sex/Gender | 1.114 | 0.0794 1.562 | 0.53 |
| Admission lactate | 1.117 | 1.064 - 1.174 | <0.0001 |
| HIV status | 1.303 | 0.828 - 2.050 | 0.25 |

**Supplemental table 4: Multivariate analysis of mortality outcome with age, lactate levels, HIV status and sex as covariates**

| **Variable** | **OR** | **95% CI** | **p-value** |
| --- | --- | --- | --- |
| **Age** | 1.004 | 0.999-1.009 | 0.092 |
| **Sex** | 1.131 | 0.776-1.652 | 0.52 |
| **Lactate** | 1.100 | 1.043-1.160 | <.001 |
| **HIV status** | 1.222 | 0.836-2.937 | 0.57 |

Supplemental Figure 1
